# Tracking the origins and drivers of subclonal metastatic expansion in prostate cancer

**DOI:** 10.1038/ncomms7605

**Published:** 2015-04-01

**Authors:** Matthew K.H. Hong, Geoff Macintyre, David C. Wedge, Peter Van Loo, Keval Patel, Sebastian Lunke, Ludmil B. Alexandrov, Clare Sloggett, Marek Cmero, Francesco Marass, Dana Tsui, Stefano Mangiola, Andrew Lonie, Haroon Naeem, Nikhil Sapre, Pramit M. Phal, Natalie Kurganovs, Xiaowen Chin, Michael Kerger, Anne Y. Warren, David Neal, Vincent Gnanapragasam, Nitzan Rosenfeld, John S. Pedersen, Andrew Ryan, Izhak Haviv, Anthony J. Costello, Niall M. Corcoran, Christopher M. Hovens

**Affiliations:** 1 Department of Surgery, Division of Urology, Royal Melbourne Hospital and University of Melbourne, Parkville 3050, Victoria, Australia; 2 The Epworth Prostate Centre, Epworth Hospital, Richmond 3121, Victoria, Australia; 3 Centre for Neural Engineering, Department of Computing and Information Systems, University of Melbourne, Parkville, Victoria 3010, Australia; 4 Cancer Research UK Cambridge Institute, University of Cambridge, Cambridge CB2 0RE, UK; 5 Diagnostic Genomics, NICTA, Victoria Research Laboratory, The University of Melbourne, Parkville, Victoria 3010, Australia; 6 Cancer Genome Project, Wellcome Trust Sanger Institute, Hinxton CB10 1SA, UK; 7 Department of Human Genetics, KU Leuven, Herestraat 49 Box 602, B-3000 Leuven, Belgium; 8 Cancer Research UK London Research Institute, London WC2A 3LY, UK; 9 Academic Urology Group, Addenbrookes Hospital, Cambridge University, Hospitals NHS Foundation Trust, Cambridge Biomedical Campus, Hills Road, Cambridge CB2 0QQ, UK; 10 Centre for Translational Pathology, University of Melbourne, Parkville 3050, Victoria, Australia; 11 Theoretical Division, Los Alamos National Laboratory, Los Alamos, New Mexico, USA; 12 Victorian Life Sciences Computation Initiative, The University of Melbourne, Parkville 3050, Victoria, Australia; 13 Department of Radiology, Royal Melbourne Hospital, Parkville 3050, Victoria, Australia; 14 Department of Histopathology, University Cambridge Hospitals, Addenbrookes Hospital, Hills Road, Cambridge CB2 0QQ, UK; 15 TissuPath Specialist Pathology, Mount Waverley 3149, Victoria, Australia; 16 Monash University Faculty of Medicine, Clayton 3168, Victoria, Australia; 17 Bar-Ilan University Medical School, Safad 1311502, Israel

## Abstract

Tumour heterogeneity in primary prostate cancer is a well-established phenomenon. However, how the subclonal diversity of tumours changes during metastasis and progression to lethality is poorly understood. Here we reveal the precise direction of metastatic spread across four lethal prostate cancer patients using whole-genome and ultra-deep targeted sequencing of longitudinally collected primary and metastatic tumours. We find one case of metastatic spread to the surgical bed causing local recurrence, and another case of cross-metastatic site seeding combining with dynamic remoulding of subclonal mixtures in response to therapy. By ultra-deep sequencing end-stage blood, we detect both metastatic and primary tumour clones, even years after removal of the prostate. Analysis of mutations associated with metastasis reveals an enrichment of *TP53* mutations, and additional sequencing of metastases from 19 patients demonstrates that acquisition of *TP53* mutations is linked with the expansion of subclones with metastatic potential which we can detect in the blood.

## Abstract (web summary)

Primary prostate tumours are known to be genetically heterogeneous and clonal selection has the potential to drive metastasis. Here Hong *et al*. show that the acquisition of TP53 mutations is linked to clonal expansion and metastatic progression to lethality.

## Graphical abstract

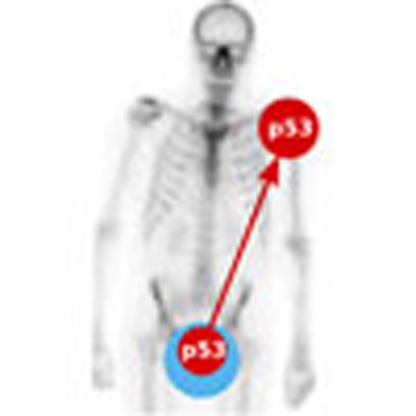

Prostate cancer in the Western world represents a continuing clinical paradox. The prostate is the most cancer-prone internal organ based on cancer incidence rates^1^, yet only an unpredictable 10% of prostate cancer cases progress to lethality. Similar to most other solid tumours, nearly all lethal cases segregate with metastasis and subsequent emergence of therapy-resistant disease. Large-scale genomic analyses have been reported for primary localized prostate cancer^1^,^2^,^3^,^4^ as well as for metastatic end-stage cancers^5^,^6^,^7^. However, these studies have been restricted to reporting the most prevalent somatic aberrations associated with the dominant clone of the tumour without permitting an analysis of subclonal complexity or how this complexity impinges on metastatic potential and resistance to treatment. Studies using exome sequencing^8^ or low-resolution genomic sequencing^9^ of primary and metastatic tumours in pancreatic cancer were some of the first to explore the clonal relationships between primary and metastatic tumours. Despite their small patient cohorts, these studies revealed seminal processes in metastatic progression including the identification of founder mutations and the timing of metastatic progression^8^, varying phylogenetic relationships between primary and metastatic neoplasms and organ-specific signatures for metastatic subclonal branches^9^. More recent studies highlight the potential of deep genomic analysis and multiregion sequencing of primary tumours from small cohorts of patients for exploring the nature of intratumour heterogeneity, and for discovering genomic processes linked with cancer evolution^10^,^11^. To identify the origins of candidate subclones contributing to metastasis and decrypt drivers of tumour subclonal expansion, in-depth longitudinal genomic studies are required^10^.

By performing whole-genome and ultra-deep targeted sequencing of longitudinally collected primary and metastatic tumours we are able to observe the direction and timing of metastatic spread. We show that a distant bony metastasis caused a local recurrence and not the other way round. We observe for the first time cross-metastatic site seeding combining with dynamic remoulding of subclonal mixtures in response to therapy. We can detect the presence of metastatic and primary tumour clones in blood, even years after removal of the prostate. Finally, by analysing mutations associated with metastasis we demonstrate that acquisition of *TP53* mutations is linked with expansion of subclones with metastatic potential and these mutations can be detected in the blood.

## Results

### Mapping tumour and metastatic subclonal heterogeneity

To map the trajectory of subclonal expansion and trace the origins of metastatic subclones, we performed longitudinal and multiregion sampling yielding 26 samples across four patients with lethal prostate cancer. Samples were collected from primary tumours and then longitudinally after the emergence of distinct metastatic foci, facilitating exploration of the genetic changes associated with metastasis. Fresh samples were interrogated with whole-genome sequencing (WGS), RNA sequencing and single-nucleotide polymorphism (SNP) profiling. Formalin-fixed, paraffin-embedded (FFPE) samples (as well as the fresh samples) were also interrogated with deep, targeted resequencing of variant loci identified from the WGS data (Supplementary Fig. 1). In addition to these four patients, extra metastases from another three patients with advanced disease were interrogated using WGS to assess drivers of subclonal and metastatic expansion, (results summarized in Supplementary Table 1).

**Figure 1:**
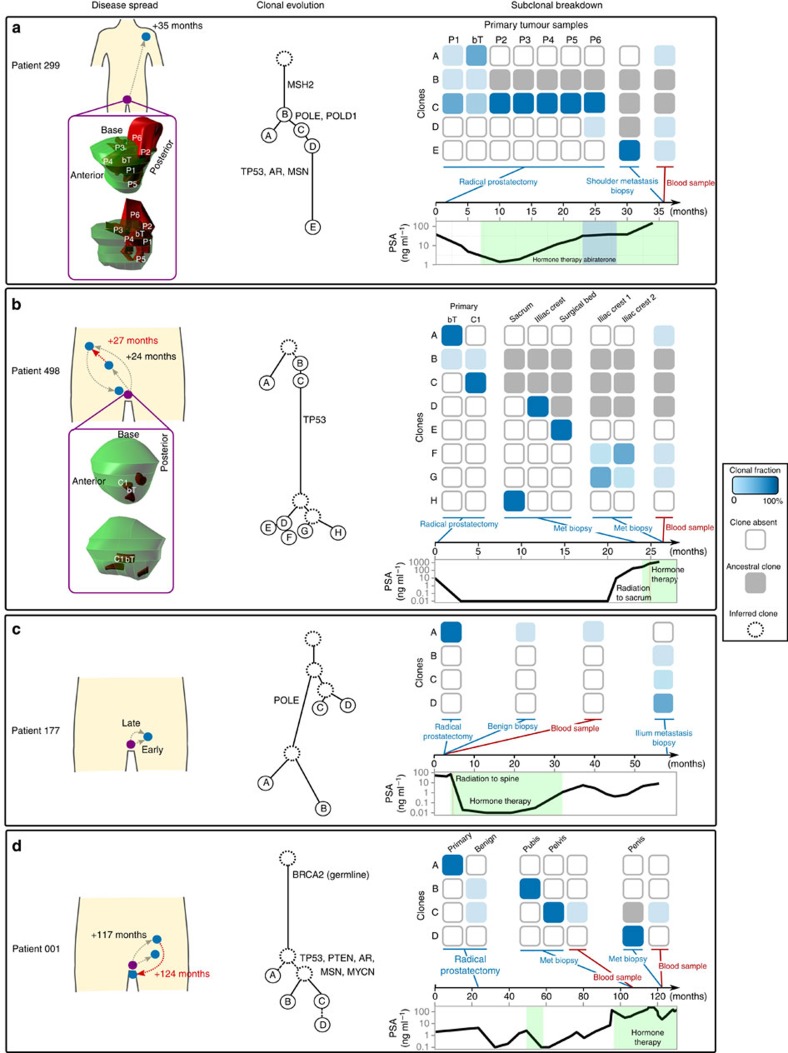
Diagrams of patient sampling and patterns of metastasis for four patients. In each of the panels, the diagram to the left depicts the timing and direction of metastatic spread for patients 299 (**a**), 498 (**b**), 177 (**c**), 001 (**d**). For patients 299 (**a**) and 498 (**b**), the multiple regions of the primary tumour that were sampled are indicated in the 3D models of the prostate reconstructed from prostate cross-sections. In the centre of each panel is an evolutionary tree depicting the distinct clones identified during the evolution of the tumour. The branch length is approximately proportional to the number of structural variations and SNVs. The matrix plots at the right of each panel represent the percentage of each clone in a given sample. The plot below this represents the disease progression for each patient measured by levels of prostate-specific antigen (PSA) along with an indication of when tumour or blood specimens were sampled and information on treatment phases.

In contrast to previous studies, which took an archaeological approach to computationally reconstruct the evolution of a tumour from bulk sequencing of a sample at a single time point^12^, we reconstructed tumour evolution using samples from multiple time points. By directly observing the genomic changes during disease progression, we could discern the exact patterns of metastatic spread (Fig. 1). To enhance our reconstruction approach, we selected mutations representative of different clones identified from the WGS data, and interrogated these mutations using deep targeted sequencing in additional samples (26 in total) across the four patients (average sequencing depth of 2,385 × , see Supplementary Fig. 1 for a workflow overview). This approach revealed a number of interesting patterns of disease spread outlined below.

### Spatial spread is linked with late arising clones

Fresh tissue of patient 299 from the prostatectomy specimen and castration-resistant shoulder metastasis sampled 35 months later were subjected to WGS along with matched whole blood. Using mutation clusters identified from initial tumour evolution reconstruction (see Methods, Supplementary Fig. 2a), a further six regions of the primary tumour (FFPE) along with the original primary, metastasis and whole-blood samples were subjected to deep targeted sequencing and detailed evolution analysis. The resulting tumour evolution tree (Fig. 1a) shows branching early in the disease, with a further linear pattern of evolution after metastasis. The original index primary tumour sample (bT) showed evidence of subclonality, and consisted of two distinct clones of which the minor clone (36%) gave rise to the metastasis. Two other primary samples exhibited subclonality: P1, from the centre of the prostate, composed of the ancestral clone (B, Fig.) along with a mixture of the two child clones (A and C, Fig. 1a) found in the primary; and P6, a sample from the seminal vesicle composed of a clone related to the metastasis (C, Fig. 1a), and a more evolved clone (D, Fig. 1a). The remaining tumour samples (P2–5, Fig. 1a) were clonal, consisting of the clone that gave rise to the metastasis (C, Fig. 1a). Interestingly, when observing the location of these samples in three dimensions (Fig. 1a), samples towards the centre of the prostate (bT and P1, Fig. 1a) were composed of less evolved clones, whereas the samples towards the outside of the tumour were made up of more advanced clones. Sample P6, containing the most advanced primary clone extended into the seminal vesicle and most likely gave rise to the population of cells that seeded the shoulder metastasis.

### A distant metastasis can reseed the surgical bed

For patient 498, WGS was completed on samples from three phases of the disease: on the primary tumour obtained at prostatectomy (bT); later on a local recurrence (surgical bed), iliac crest metastasis and sacral metastasis before systemic treatment; and again on the same iliac crest metastasis after treatment failure and emergence of castration resistance, (Fig. 1b). Deep targeted sequencing (2,076 variants Supplementary Fig. 2b) was also performed on these samples, along with an additional FFPE primary sample and end-stage whole blood.

Evolutionary reconstruction showed that the index primary tumour was largely unrelated to the metastatic samples, with only a minor clone containing any mutations shared with the clone that give rise to the metastases (clone B, Fig. 1b, Supplementary Figs 2b and 7b). Profiling of the FFPE tumour, which was sampled from the same focus as the index tumour, showed a different subclonal makeup, with the bulk of the sample consisting of the clone ancestral to the metastases (clone B, Fig. 1b).

The first striking observation from the evolutionary reconstruction was that the non-osseous local recurrence tumour within the prostatic cavity (surgical bed) was made up of a clone derived from the hormone-naïve iliac crest metastasis (clone E, Fig. 1b). While it is possible that these metastases arose within the prostate, with an early-spread seeding the iliac crest site and a late-spread seeding the surgical bed site, this would require that the iliac crest clone ceased evolving, an unlikely scenario. Therefore, the directionality of dissemination between the bony iliac crest metastasis and the local recurrence at the surgical bed is directly counter to the commonly accepted principle that local recurrences are seeded from incomplete surgical removal of localized tumours which then subsequently seed more distant sites of metastasis^13^. This raises the possibility that the surgical bed provided a niche environment for metastatic seeding.

### Cross-metastatic seeding in response to therapy

The second intriguing observation from the evolutionary reconstruction is that during the 12 weeks between sampling of the iliac metastasis site before treatment, and the same site after the development of castration resistance, dramatic remodelling of the subclonal mixture occurred. The original hormone-naive metastasis was clonal (clone D, Fig. 1b), however after treatment, the metastasis contained two distinct subclonal populations: one derived from the original clone (clone F) and a clone that appeared to be derived from the distant sacral metastasis (clone G, Fig. 1b). This second clone could have arisen either through reseeding from the distant site or it may have been present at an undetectable fraction before treatment. Nevertheless, the change in subclonal fractions suggests that the hormone-naïve iliac crest clone was sensitive to hormone therapy, while the sacral clone was already ‘hardwired’ for treatment resistance. In the case of reseeding, the iliac crest site may have served as a premetastatic niche for subsequent metastatic transfer from one metastatic site to another in response to therapy. We performed an in-depth analysis of the genomic variations between the ‘invading’ subclone and the ‘receding’ subclone at the iliac crest site. The only significant alteration we detected between the subclones was a somatic mutation in the SYK kinase gene. Loss of SYK has been associated with breast cancer progression^14^ and with poor prognosis and metastasis in a range of epithelial tumours^15^,^16^, implicating it as a potential driver of cross-metastatic seeding and treatment resistance in this patient.

### Multiple waves of metastatic seeding from the primary tumour

For patient 177, we performed WGS on the primary tumour and castration-resistant iliac metastasis harvested 57 months after prostatectomy (Fig. 1c). This patient’s tumour was dominated by structural variations early in the evolution of the tumour and as such, allele frequencies from both single-nucleotide variants (SNVs) and structural variants (SVs) had to be used to derive the initial evolutionary history for the tumour (Fig. 1c, Supplementary Fig. 3, Methods). These samples and benign tissue coupled with whole blood from radical prostatectomy, were subjected to deep targeted sequencing (145 SNVs Supplementary Fig. 2c). The evolutionary reconstruction revealed that the iliac metastasis comprised three distinct clones derived from two separate waves of metastasis from the prostate. The first spread occurred early in the evolution of the tumour yielding two of the subclonal populations making up 78% of the metastasis (clones C and D, 43+35%, Fig. 1c). These two subclonal populations shared 36 SVs and 24 SNVs suggesting that the metastatic potential was driven predominantly via structural rearrangement. The primary tumour then acquired a further 27 structural rearrangements and 1092 SNVs before a second wave of metastasis occurred. The mutations unique to this subclonal population (clone B) comprised 69 SVs but no additional SNVs suggesting that structural rearrangements were also the driver of metastatic potential in this subclone.

### A copy-number-driven tumour reveals branched evolution

Finally, for patient 001, two spatially distinct, castration-resistant metastases, one localized in the pubis and another in the pelvis were subjected to WGS (Fig. 1d). These samples, a later penile metastasis, tumour and benign tissue from radical prostatectomy, and blood collected at the time of both metastatic biopsies were subjected to deep targeted sequencing (217 variants Supplementary Fig. 2d). In contrast to the tumours from the other patients, these tumours showed a high degree of copy-number variation (Supplementary Fig. 4). In addition, the samples showed no evidence of subclonal mixtures and evolution analysis revealed a simple branching evolutionary architecture (Fig. 1d).

### Mutational signatures specific to metastasis

**Figure 2:**
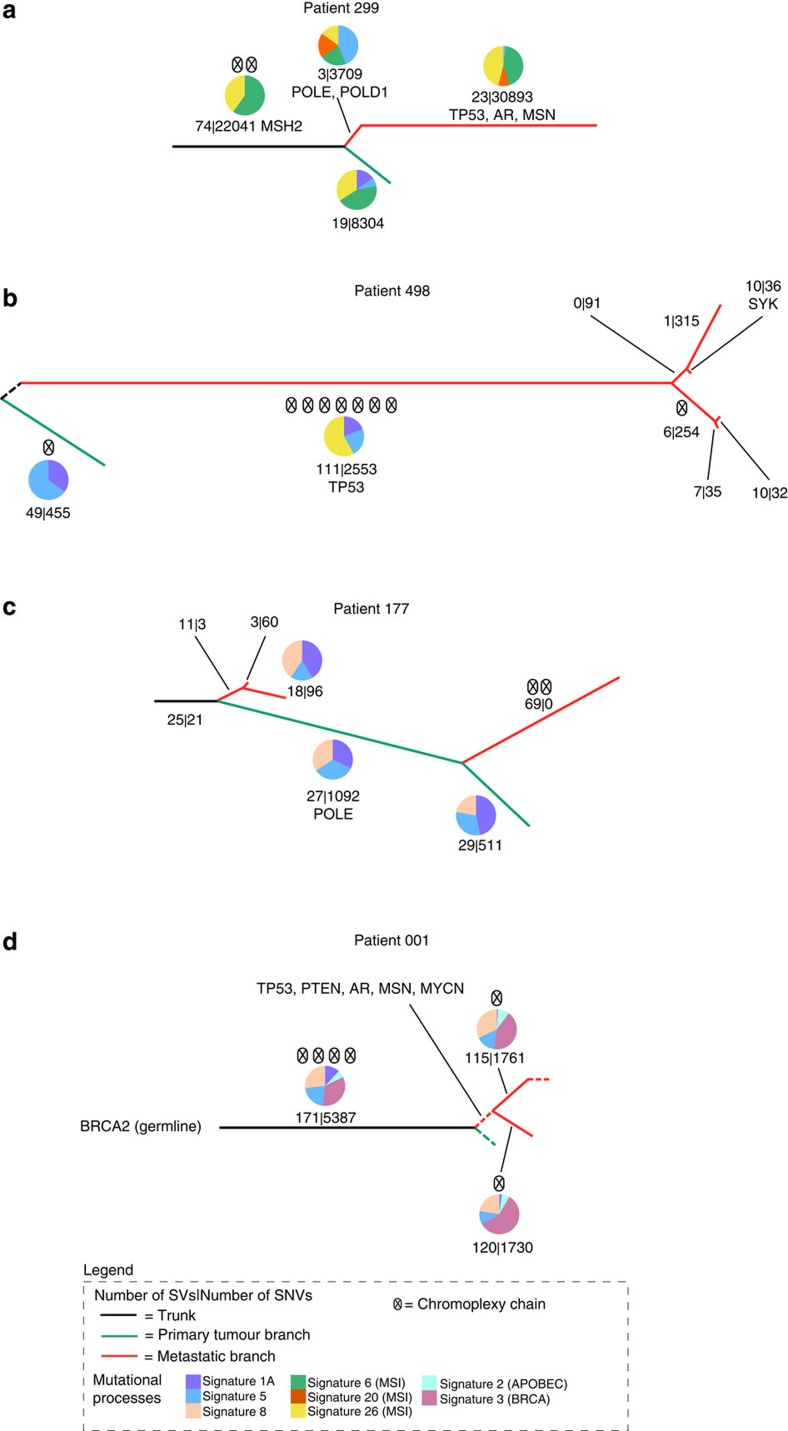
Evolution of mutational signatures and driver mutations. The trees in this diagram depict the genomic evolution of the tumours for patient (**a**) 299, (**b**) 498, (**c**) 177 and (**d**) 001. The branch lengths are approximately proportional to the number of structural variations and single-nuclotide variations. Dashed branches occur when the number of mutations could not be estimated (no WGS performed on the clone depicted in the branch). Branches are colour coded—black representing trunk (clonal) mutations, green representing the branch leading to the bulk primary tumour and red representing the branches leading to metastasis. The mutational signatures detected across the mutations in each branch are indicated via the pie charts. Signatures 1A (purple), 5 (aqua), 8 (cream), 6 (green, MSI), 20 (orange, MSI), 26 (yellow, MSI), 2 (blue, APOBEC), 3 (pink, BRCA). Chromoplexy chains are indicated via circles and crosses and mutations in known cancer drivers are listed.

A key advantage of longitudinal sample collection coupled with detailed evolutionary reconstruction is that it is possible to observe key mutation characteristic changes during tumour evolution. For each of the four patients, we performed mutation signature analysis^17^,^18^,^19^,^20^ and chromoplexy analysis^7^,^21^,^22^ using the mutations found on each branch of the trees, which allowed us to observe the role of these phenomena during the progression of these tumours to metastasis (Fig. 2). In addition, we also observed mutations in known cancer drivers.

For patient 299, the mutational processes active early on in the development of this tumour were dominated by signatures 6 and 26, both associated with microsatellite instability (MSI) and indicative of a mismatch repair (MMR) defect. This was in concordance with the large number of SNVs observed in the tumours of this patient (Supplementary Table 1). Further analysis revealed a complex SV event on chromosome 2 causing inactivation of *MSH2* which resulted in a DNA MMR defect causing the mutator phenotype observed in this patient. These observations were also supported by *MSH2* immunohistochemistry analysis (Supplementary Fig. 5). Interestingly, a third MSI signature was observed only in the branch which gave rise to the metastasis suggesting that this process may be associated with metastatic potential (signature 20 Fig. 2a). A mutation in *POLE* and *POLD1*, also residing on this branch, could account for the emergence of this signature.

For patient 498, the mutational signature analysis revealed signatures typically found in prostate cancer (signatures 1A and 5 Fig. 2b) active throughout the evolution of the tumour. An additional signature (signature 26) was detected on the metastatic branch, which is associated with MSI, raising the possibility that acquisition of a MMR defect was also associated with metastasis for this tumour. However, no mutations in known cancer genes were detected that could account for this signature.

Mutational signature analysis in patient 177 revealed signatures indicative of prostate cancer derived mutation processes (Fig. 2c), but no indication of MMR defects.

Interestingly, for patient 001, mutation process analysis revealed signatures indicating APOBEC activation (signature 2) and BRCA mutation (signature 3) as well as signatures common to prostate cancer (1A, 5 and 8), suggesting a further association with DNA-repair defects and acquisition of metastatic potential. Indeed, *BRCA2* was found to be inactivated in the metastases by a combination of a novel heterozygous germline frameshift mutation which was found in exon 11 of *BRCA2* (*g.chr13:32911991delA, p.I1167fs*) and a somatic copy-number loss of the other allele.

Mutation signature analysis on WGS of a further three patient metastases revealed two signatures, 17 and 18, which had not previously been seen in prostate cancer. Overall we observe great diversity in underlying mutational processes in these metastatic prostate samples compared with the large number of primary prostate samples previously analysed for mutational signatures^19^ suggesting greater overall diversity in the processes underlying metastases compared to tumorigenesis.

Across the samples we observed chromoplexy events occurring both early and late in the disease suggesting that chromoplexy is a key mutation process throughout the progression of the disease.

### Clones from various tumour stages are detectable in blood

The deep targeted sequencing of mutation clusters representing specific clones in the blood and benign prostate samples across four patients revealed some unexpected results. For patient 299, mutation clusters representing three clones were identified significantly above background in the blood collected at metastasis biopsy (clones A, D and E, Fig. 1a, Supplementary Fig. 8). The clone from the metastatic site was detected as expected, along with the clone representing the sample from seminal vesicle spread from the primary tumour. Unexpectedly, a clone from the primary tumour mass that did not give rise to the metastasis was also detected in the blood. This is surprising given that the blood was sampled nearly 3 years after the removal of the prostate. This suggests that all subclones from the primary were able to seed metastatic deposits and persist in clinically occult sites.

A similar case was observed for patient 498, where clones from the castrate-resistant metastasis were detected above background (clone F and G Fig. 1b), along with a clone representing the unrelated portion of the primary tumour (clone A Fig. 1b).

In patient 177, deep sequencing of blood collected at radical prostatectomy yielded clones from the primary tumour, and additional sequencing of adjacent benign prostate tissue showed low levels of primary tumour clones.

In patient 001, deep sequencing of blood at first metastasis biopsy and second metastasis biopsy revealed clones present from the pelvic metastasis, but not clones from the pubic metastasis. However, deep sequencing of adjacent benign prostate tissue from radical prostatectomy showed the presence of clones from both metastatic sites.

### Identification of drivers of subclonal expansion and spread

**Figure 3:**
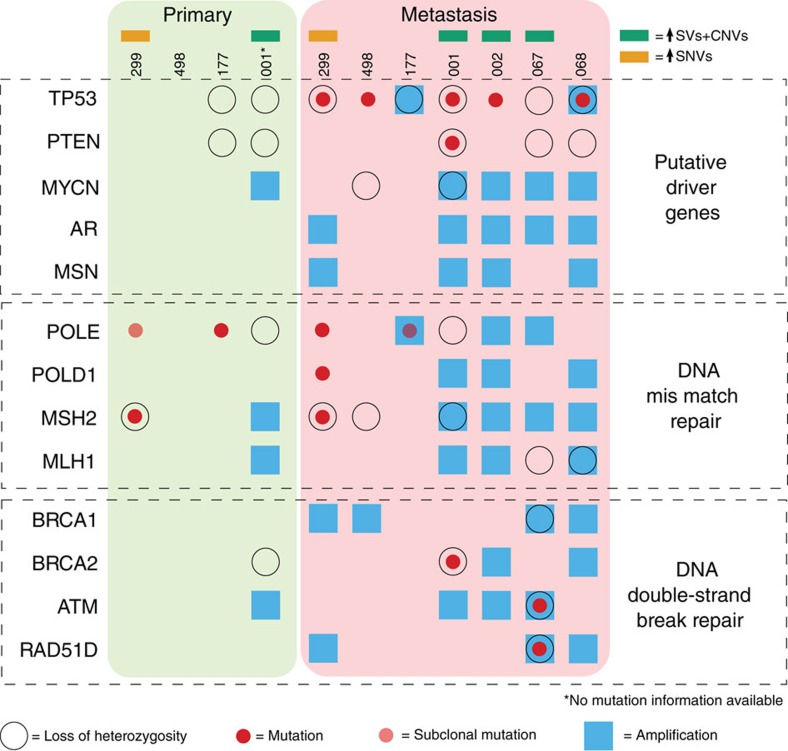
Mutations in known cancer drivers. The matrix indicates mutations identified in known cancer drivers for the primary tumours from four patients (left) and the metastases for seven patients (right).

Using all samples that underwent WGS from the four patients mentioned above, as well as WGS of an additional three metastases with matched germline (a total of 21 samples), we sought known cancer drivers that were either mutated or amplified/deleted in the metastatic samples relative to the primary tumours. Results from this analysis across the seven patients, comprising 10 metastatic and 4 primary tumours, are depicted in Fig. 3. We observed that perturbations in DNA mismatch/break repair pathway were present in all metastasis samples from the seven patients (11 in total). Perturbations in the same pathways were present in three out of four primary tumours from the same cohort. We observed a hypermutator phenotype in the metastatic and primary tumour samples from patient 299 (consistent with early loss of *MSH2*), along with metastasis-specific mutations in the *POLE* and *POLD1* DNA repair and replication genes. Specific germline mutations in *POLD1* and *POLE* have recently been shown to cause multiple colorectal and endometrial tumours in affected individuals^23^,^24^, and the mutation in *MSH2* is consistent with the observation that MMR gene mutations are enriched in high-grade prostate cancers^25^. For metastatic samples derived from patients 001 and 067, we detected a DNA instability hypermutator phenotype consistent with mutations in the *BRCA2* and *ATM*/*RAD52* homologous recombination repair genes. In addition, we observed a strong recombination repair deficiency signature (Supplementary Fig. 4) in the metastatic genome of patient 002, without being able to discern a mutation in any known DNA repair pathway gene. Across all four metastatic samples from patient 498, we detected a clear MSI signature (Fig. 2), without being able to ascribe this genotype to a specific MMR pathway.

At the depth of WGS we performed (average 48 × ), we observed a marked enrichment in *TP53* mutations that were restricted to metastases, with five out of seven patients exhibiting *TP53* defects and none found in the matching primary tumours. Only one metastasis had a mutation in the *PTEN* tumour suppressor gene. Amplification of the AR and MycN loci was also highly enriched in the metastatic samples (Fig. 3).

**Figure 4:**
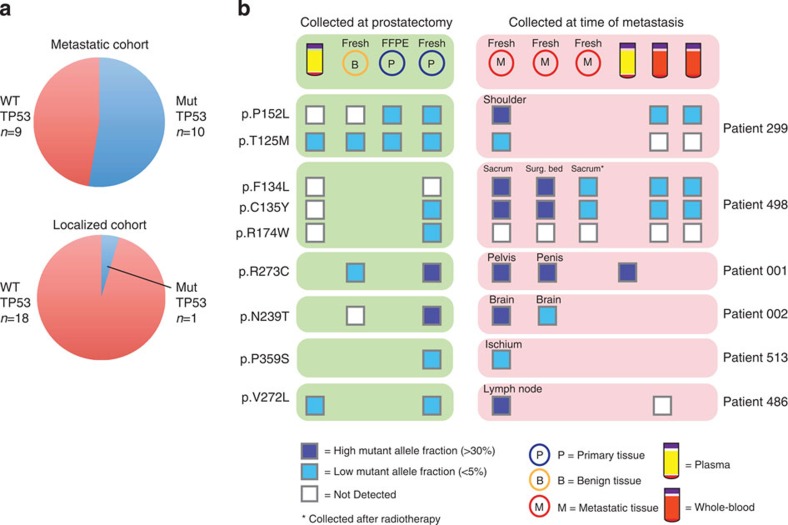
TP53 mutations identified via TAm-Seq. (**a**) Pie charts representing the number of patients with detected TP53 mutations across the metastatic and localized cohorts. (**b**) A schematic indicating the presence of TP53 mutations and their allele frequency for all patients in the metastatic cohort that had matched primary and metastatic samples. Surg bed, surgical bed.

The high frequency at which the TP53 mutations appeared in the metastases from WGS data suggested that TP53 mutations were clonal in the metastases. Conversely, the lack of *TP53* mutations in the matching primary tumours suggested that either the *TP53* mutations were not present or present in low-frequency subclones. To explore this further, we analysed an additional 12 patients with metastatic prostate cancer, five of whom had paired primary tissue samples, and an additional 19 patients with localized disease, performing tagged-amplicon deep sequencing (TAm-Seq^26^) on all 38 patients to interrogate entire exonic regions of *TP53*. This combined cohort of 19 metastatic and 19 localized patients comprised 91 samples, including 48 metastatic tissue/blood/plasma samples and 43 primary tissue/blood/plasma samples. Each sample was sequenced to an average depth of 7,848 × permitting detection of low-frequency *TP53* mutations. The results (summarized in Fig. 4, Supplementary Table 2 and Supplementary Fig. 9) show that 10 out of 19 metastatic patients had detectable *TP53* mutations, compared with one out of the 19 patients with localized disease. The increased depth of targeted sequencing over WGS surprisingly showed that in the six out of the 10 metastatic patients for which we had a matching primary sample, the primary tumours had *TP53* mutations appearing mostly at low frequencies (Fig. 4b). Furthermore, in most of these cases, we were able to detect these mutations in the blood or plasma.

This enrichment of *TP53* mutations in the metastasis samples, which appear at low frequency in primary samples, strongly suggests that the acquisition of *TP53* mutations in the prostate cancer setting enhances the metastatic potential of tumour subclones. Further supporting this observation is the secondary *TP53* mutations found in patients 299 and 498. In both patients, a *TP53* mutation was detected at high frequency in the metastases, and this mutation was detected at low frequency in the primary. However, a second *TP53* mutation was detected at low frequency in the primary tumour, which was not found at high frequency in the metastasis. If these low frequency, secondary *TP53* mutations in the primary tumour confer metastatic potential, then the clones containing this mutation should be more likely to be detected in the blood/plasma of these patients. At the depths of sequencing we achieved with TAm-Seq analysis, we were unable to detect these mutants in the whole blood specimens; however, the deep mutation-specific sequencing we performed using the Nextera capture did permit the detection of blood-specific metastatic clones that were unique to the primary tumour 3 years after removal of the prostate, strongly suggesting that the acquisition of a separate *TP53* mutation in these blood-specific clones led to their ability to metastasize (Fig. 1).

## Discussion

The combination of longitudinal sampling coupled with multiregional probing of primary tumours has revealed an integrated picture of the clonal diversity across spatial and temporal dimensions within individual patients.

Deep analysis of samples from a single patient has revealed cross-metastatic site seeding in direct response to treatment. A candidate driver of this process has been identified in the *SYK* oncogene. This suggests that certain individual metastases in multi-metastatic patients have the potential to seed and reseed other metastatic niches. This would imply that certain ‘index’ metastases are more appropriate targets for therapeutic intervention and that individual metastases cannot be regarded as clinically equivalent.

We have also observed that multiple, temporally separated waves of metastasis can occur from the primary tumour, implying that surgical removal of the primary tumour may be warranted even from advanced cases. Our analysis has also enabled us to determine the direction of metastatic spread in detail, exemplified by a local recurrence tumour at the primary site reseeded by a distant bone metastasis and not vice versa (patient 498).

At the present time, we cannot assess the prevalence of these phenomena in the general advanced prostate cancer population as these observations are for single patients. Further larger cohort studies are required to assess the incidence of these findings in the general disease setting. However, such observations cannot be made from cancer genomics studies that focus on large cohorts of samples from single time points^1^,^2^,^3^,^4^,^5^,^6^,^7^ or from limited analysis of variant regions in advanced cases^27^.

The late acquisition of *TP53* missense mutations in low-frequency subclones in the primary tumour appears to be the driver for metastasis since we detect identical *TP53* mutations at high frequency in the matching metastases and furthermore can detect these mutations in the blood. Missense *TP53* mutations appear to confer a gain-of-function activity, distinct from *TP53* null mutations, which leads to increased metastatic incidence in mouse carcinoma models^28^,^29^ and confers a poor prognosis in breast cancer patients^30^. The incidence of *TP53* mutations in primary prostate cancer has been reported as much lower than other carcinomas such as pancreatic, ovarian, breast or colon cancers, with higher frequencies detected in prostate cancer metastatic samples^31^, though these results have been disputed^32^. Our results suggest that the late acquisition of *TP53* missense mutations in the context of a rearranged genome may confer metastatic potential to primary tumour subclones and that these events may be detectable in the blood. These findings now need confirmation in expanded, paired longitudinal studies.

In addition, mutational process analysis has revealed different MSI signatures active only in the metastatic phase of the disease in contrast to primary tumours where the repertoire of such signatures is more limited. This difference in underlying DNA-repair defect suggests the potential for this process to drive metastatic subclonal expansion. Such findings may have implications for lethal prostate cancer subtype classification and stratification, hypotheses requiring further testing in large independent series.

Our findings reveal complexity in the patterns of metastatic dissemination as well as candidate mutational processes and genes driving metastatic expansion in lethal prostate cancer. These results now imply that, notwithstanding the heterogeneity known to be present in primary tumours^32^,^33^,^34^,^35^,^36^,^37^, metastatic subclones might be able to be detected and tracked both in tumour tissues and in the blood.

## Methods

### Patient information

For the metastatic cohort, following informed consent from all patients and institutional ethics approval from the Royal Melbourne Hospital Human Ethics committee, suitable prostate cancer patients were invited to undergo voluntary biopsy of their metastases^38^. This metastatic selected clinical cohort comprised primary adenocarcinomas spanning the common clinical grades and stages ranging from Gleason scores of 7 to 9, pathological T stage 2c, 3a, 3b and prostate-specific antigen levels on prostate cancer diagnosis from 4.4 to 52.9.

For the localized cohort, localized prostate cancer patients were recruited in Addenbrookes Hospital, Cambridge, and informed consent obtained as part of the on-going ethically approved PROMPT trial, ethics approval granted by the Trent Multi-Centre Research Ethics Committee. The localized cohort consisted of primary adenocarcinomas ranging from Gleason 6–9, pathological T stage 2c, 3a, 3b and prostate-specific antigen at diagnosis from 2.86 to 89. To date, no cases have developed metastatic symptoms or biochemical recurrence despite median follow up of 3 years.

### Tissue procurement and processing

For metastatic samples^38^, a coaxial bone biopsy needle with an 18G internal calibre (Bonopty Bone Biopsy System, 10-1072, AprioMed AB, Sweden) was used and tissue cores were immediately placed in a 1.5-ml microcentrifuge tube and flash frozen in liquid nitrogen. A 14G needle (Quick-Core Disposable Biopsy Needle, QC-14-9.0-20T, Cook, Brisbane, Australia) was used for ultrasound guided biopsies if deemed clinically appropriate. Samples were also flash frozen in liquid nitrogen and transported on dry ice. Long-term storage was in liquid nitrogen vapour phase. Tissue samples were embedded in optical cutting time compound (Sakura) at −24 °C and 5-μm sections cut by cryotome (CM1,900, Leica Microsystems, NSW, Australia). Sections were transferred to charged glass slides (Superfrost Ultra Plus, Thermo Scientific), rapidly stained with haematoxylin and eosin, and assessed in real time by a pathologist for tumour content. On confirmation of malignancy or where this was considered likely but inconclusive, the optical cutting time compound-embedded tissue samples were isolated with a scalpel and placed in 700 μl RLT Plus buffer for immediate homogenization (TissueRuptor, Qiagen, CA). DNA and RNA were simultaneously extracted using the Allprep Micro Kit (Qiagen, CA) following manufacturer’s instructions and including on-column DNAse digestion of the RNA. Genomic DNA was extracted from fresh frozen samples of whole blood with the DNeasy Blood & Tissue Kit (Qiagen, Maryland) following the manufacturer’s instructions. RNA quantity and quality were checked by microelectrophoresis (Agilent 2100 Bioanalyzer), while DNA quantity was checked by spectrophotometry (NanoDrop 1000, Thermoscientific) and the quality was assessed by gel electrophoresis (0.8% agarose gel).

### SNP array

Genomic DNA was prepared using the Infinium HD assay ultra workflow (Illumina) and hybridized onto the Illumina HumanOmni2.5 BeadChip or the Illumina HumanOminExpress BeadChip following the manufacturer’s instructions. In brief, the DNA was normalized to 200 ng in 4 μl of water, enzymatically fragmented and repurified. The library was then hybridized onto the BeadChip, which was scanned on an iScan machine (Illumina) to generate intensity values.

### 3D tumour reconstruction

We converted the horizontal two-dimensional tumour maps consisting of horizontal sections taken 5 mm apart from base to apex, with vertical sections taken at varying thicknesses at the base and apex into three-dimensional (3D) models using the 3D reconstruction programme Rhinoceros v5. Two-dimensional prostate images were converted to separate prostate and tumour paths using Adobe Photoshop then exported into Rhinoceros for image stacking, alignment and lofting to create a stylized 3D view of prostate and tumour surfaces.

### Whole-genome sequencing

*DNA library preparation*. One μg of genomic DNA was used as input into the TruSeq DNA Sample Preparation Kit v2 (Illumina, FC-121-2001) and processed according to the manufacturer’s instructions. In brief, genomic DNA was sheared by ultrasonication (Covaris S2 fragmentation machine) with the following settings: duty cycle 10%, intensity 5.0, 200 cycles per burst, 40 s duration, frequency sweeping mode, 23 W and 5.5 to 6 °C. The fragmented DNA was end repaired, the 3′ ends adenylated and indexed paired-end adaptors ligated. Following purification, the libraries were run on a 2% agarose gel on the Pippin Prep (Sage Science) electrophoresis platform and 300–400 bp insert size selection performed (at a location corresponding to 400–500 bp to account for the size of the ligation adaptors). DNA purification was performed using the MinElute Gel Extraction Kit (Qiagen) and the library amplified by PCR to enrich for DNA fragments with both ends ligated with adaptor molecules.

*Quality control*. One μl of the resuspended construct was loaded on an Agilent Techologies 2100 Bioanalyzer using an Agilent DNA-1000 chip, a DNA specific chip. The final product was checked for a size of 300–400 bp.

*Normalization and pooling*. Ten μl of the sample library was normalized to 10 nM using Tris-Cl 10 mM, pH8.5 with 0.1% Tween 20. Samples were multiplexed with multiple samples per lane in the flow cell, and were thus pooled accordingly.

Sequencing cluster generation occurred on a cBot automated cluster generation system using TruSeq PE Cluster Kit v3 (cBot-HS, Illumina) reagents for 100-bp paired-end sequencing. Each flow cell was loaded onto a HiSeq2000 sequencing platform with reagents from TruSeq SBSv3 HS (200 cycles; Illumina) and 120 to 180 million reads per flow cell lane performed.

### Custom capture sequencing

We employed Illumina’s Nextera Custom capture technology to enrich for 561 variants for patient 299, 2,076 variants for patient 498, 145 variants for patient 177 and 217 variants for patient 001. These variants were selected from those identified from WGS of the fresh frozen tumours. Variants were prioritized based on their proximity to heterozygous germline SNPs. This assisted in phasing of the variants, as each read contained a somatic variation and germline variation, allowing accurate estimates of copy number and allele frequency. The capture was applied to all fresh frozen tumour samples, matched whole blood for each patient and additional FFPE samples (summarized in Supplementary Fig. 1).

Custom capture assay and probe design was performed using the Illumina Design Studio online software tool. A first iteration of the assay using Nextera Custom Capture technology failed to achieve target enrichment rates over 5%, while capturing >80% repeat regions. A second iteration of the assay, designed to specifically exclude low-specificity regions and utilizing the improved Nextera Rapid Capture technology yielded satisfactory data for all processed samples.

Samples were processed in batches of 12 per capture before sequencing on Illumina MiSeq (QC only) or HiSeq 2500 Rapid sequencers. In brief, tagmentation, initial amplification and barcoding of individual samples were performed according to manufacturer’s instructions using 50 ng of input DNA. Success of tagmentation and quality of post-amplification libraries were assessed using BioAnalyzer (Agilent) and Qubit hsDNA fluorimetric quantitation (Invitrogen). For each sample, 500 ng of barcoded library were added to the precapture pool, which was subsequently concentrated to a total volume of 40 μl using Amicon Ultra centrifugation columns. Probe hybridization was performed twice over night (Nextera Custom Capture) or for 90 min and overnight (Nextera Rapid Custom Capture), followed by capture, wash and clean-up according to the manufacturer’s instructions. After the second round of library amplification, quality of post-capture pools was again assessed using Qubit and BioAnalyzer. Before sequencing, libraries were diluted to 8 pM and denatured according to the manufacturer’s instructions. Two capture pools containing libraries from 12 samples each were then sequenced on a single HiSeq 2500 Rapid flowcell, producing ~130 × 10^6^ paired 2 × 100 base-pair reads. For the Nextera Rapid Custom Capture samples, an average 60% of reads were in the desired target regions, producing sufficient high-quality data for subclonal analysis.

After sequencing, each variant had on average a coverage of 2,385-fold. To detect and remove any potential artefacts, we carried out the following procedure. All reads with base quality or read quality <30 were removed. To ensure that no cross-contamination occurred between samples, we tested to see if any variants showed a monotonically increasing or decreasing allele frequency between at least four neighbouring samples on the capture, which may be suggestive of diluted cross-contamination, no such relationships were found. All variants showing all four bases at similar frequencies were consider artefacts and removed.

We also employed the following procedure to determine the sensitivity at which we could accurately detect subclonal populations. For fresh frozen and FFPE samples independently, we took all variants showing non-zero read depths across all samples in a batch (fresh=259, FFPE=490). For each of these variants, we excluded the samples from the patient the variant was called in and recorded the maximum frequency observed across all remaining samples. We then used the mean and s.d. of these maximum frequencies to determine a threshold for reliably calling frequencies—mean+3 × s.d. (fresh=0.0054, FFPE=0.0137).

### DNA read alignment and quality control

We performed read alignment using the Genome Analysis Toolkit protocol recommendations for variant detection and quality control (v4) ( http://www.broadinstitute.org/gatk/guide/topic?name=best-practices).

### Somatic SNV calling

We ran two independent variant callers, Mutect^7^ and Somatic Sniper^39^ and only considered variants called by both methods to be putative somatic variations. To estimate the false positive call rate using this approach, we relied on our deep RNA sequencing. We considered all tumour samples from patient 498, WGS coverage (20–31 × ), tumour purities (23–82) and looked for regions that had at least a read depth of 10 RNA-SEQ reads. Using these variants, we were able to determine true positive calls (those that appear in both DNA and RNA) and false positive calls (those appearing in DNA but not RNA). At the default score thresholds for each algorithm, we calculated an average precision of 89.7% across the four samples. This was below our desired 95% precision. To increase this, we adjusted the Somatic Sniper Score threshold to provide an average precision >95% (>30). Variants discovered across all tumours from the same patient were recalled in each sample (final recall 92%). Mutect’s t_lod_fstar score had a satisfactory average precision of 94% with default settings. To ensure no germline artefacts were present, all variants were filtered against dbsnp138 (ref. 40) and complete genomics 69 genome variants^41^. To further remove artifacts, we filtered the variants against simple repeats annotated using repeatMasker. We estimated the validation rates using variant allele frequencies from the high-depth Nextera Custom Capture sequencing. A variant was taken to be validated if the called alternate allele was supported by at least 20 reads and at least 0.5% of reads in the Nextera data. These thresholds were chosen to allow validation of low-frequency variants, while keeping the calling threshold above the observed noise level in the Nextera data. Validation rates across samples, using all variants, ranged from 95.6 to 99%. However, when variants with a Nextera read depth of <200 were excluded, validation rates rose to 99.9–100%, indicating that these variants may have failed to validate due to insufficient coverage in the Nextera capture.

### Somatic structural variation calling

Socrates^42^ was used to detect genomic breakpoints in matched whole blood and tumours for each patient. All breakpoints found in whole-blood samples were used to filter out putative germline breaks. All breakpoints that were <100 bp were removed. We determined a threshold for read depth using the following approach: the tumour read depth for each sample was calculated as *p* × *d* where *p*=tumour purity and *d*=average sequencing read depth. We aimed to detect SVs exhibited in at least 5% of tumour cells and therefore the read depth threshold used was 0.05 × *p* × *d* (rounded to the nearest integer). SVs having long soft-clipped (>25 bases clipped) read depths less than the threshold at both ends were removed. SVs having the number of spanning read pairs less than the threshold were also removed. Spanning read pairs that were considered were those which had one of the read pairs aligning either side of the breakpoint, not soft-clipped and whose insert size was within 3 × s.d. of the average insert size for the sample. Any structural variations that had an average MAPQ score across all of the soft-clipped reads <20 were removed. Any structural variations that had both ends mapping to simple repeats or satellite repeats were considered likely artefacts and removed. To remove any artefacts resulting from regions with unusually high normal read coverage, we placed a threshold of 0.01 on the tumour allele fraction, that is, the no. of reads supporting break/(no. of normal reads+no. of reads supporting the break) >0.01. Finally, all SVs were manually inspected using the Integrated Genomics Viewer and suspect SVs removed.

### Somatic copy-number calling

For SNP array data, ASCAT^43^ was used to estimate copy number, tumour purity and ploidy. For WGS data, the Battenberg algorithm^12^ was used to call copy number, tumour purity and ploidy. In brief, the Battenberg algorithm uses Impute2 (ref. 44) to phase heterozygous SNPs with use of the 1,000 genomes genotypes as a reference panel^45^. The resulting haplotypes are corrected for occasional errors in phasing in regions with low linkage disequilibrium. After segmentation of the resulting B-allele frequency values, *t*-tests are performed on the BAFs of each copy-number segment to check whether they correspond to the value resulting from a fully clonal copy number change. If not, the copy-number segment is represented as a mixture of two different copy-number states, with the fraction of cells bearing each copy-number state estimated from the average B-allele frequency of the heterozygous SNPs in that segment.

### TAm-Seq analysis for TP53 mutations

DNA was extracted from fresh frozen tissue, FFPE tissue and whole-blood samples using the DNeasy Blood and Tissue kit according to the manufacturer’s protocols. One ml of plasma and 200 μl of selected whole-blood samples were extracted using QIAamp circulating nucleic acid and QIAmp mini blood kits (Qiagen), respectively. Genomic libraries were prepared as previously described^26^. Libraries were quantified with KAPA qPCR library quantification kit, and sequenced on a MiSeq Sequencer (Illumina) using 150 bp paired-end sequencing protocol over two lanes. Reads were demultiplexed according to sample-specific barcodes and aligned to the reference genome (hg19) with BWA (0.7.5a)^46^. Mutations were called and quantified as previously described^26^.

### RNA sequencing

*cDNA library preparation*. One μg total RNA was used as input into the TruSeq RNA Sample Preparation Kit v2 (Illumina, RS-122-2001) according to manufacturer instructions. In brief, poly-A RNA was purified using poly-T oligo-attached magnetic beads, then fragmented using divalent cations under elevated temperature. Random hexamers for reverse transcriptase priming were added and the cleaved RNA fragments copied into the first-strand complementary DNA (cDNA). Second-strand cDNA synthesis was performed using DNA Polymerase I and RNase H. The resulting cDNA fragments were end repaired, 3′ ends adenylated and indexed paired-end adaptors ligated. The products were purified and then enriched with PCR to create the cDNA library.

*Quality control*. One μl of the resuspended construct was loaded on an Agilent Techologies 2100 Bioanalyzer using an Agilent DNA-1000 chip. The final product was checked for an approximate size of 260 bp.

*Normalization and pooling*. Ten μl of the sample library was normalized to 10 nM using Tris-Cl 10 mM, pH8.5 with 0.1% Tween 20. Samples were multiplexed with multiple samples per lane in the flow cell and were thus pooled accordingly.

*Sequencing*. Cluster generation occurred on a cBot automated cluster generation system using TruSeq PE Cluster Kit v3 (cBot-HS, Illumina) reagents for 100-bp paired-end sequencing. Each flow cell was loaded onto a HiSeq2000 sequencing platform with reagents from TruSeq SBSv3 HS (200 cycles; Illumina) and 120 to 180 million reads per flow cell lane performed.

### RNA read alignment and quality control

Raw sequence data was clipped for Illumina adapter sequences using Trimmomatic^47^. Reads were aligned to the 1,000 genomes hg19 build 37 reference annotation using Tophat2 v2.0.4 (ref. 48) with default parameters using Bowtie v0.12.8 (ref. 49).

### Identification of regions of chromoplexy

For each evolutionary hierarchy, every final descendant (that is, having ancestors but not descendants) was interrogated iteratively for chromoplexy events using ChainFinder^7^ (*P* value threshold of 0.1) using variations identified by Socrates and CNVs identified by ASCAT. With a bottom-up approach, every ancestor was annotated with the number of rearrangements present in the ‘rearrangements chain’ of all its final descendants.

### Mutational process analysis

Recently, 27 distinct consensus signatures of mutational processes from 7,042 samples across 30 different cancer types were identified^19^. We used these consensus mutational signatures, plus a number of unpublished signatures (including signature 26) and our previously developed computational framework^50^ to evaluate the set of signatures that optimally explain the somatic mutations in each of the prostate cancer samples. All possible combinations of at least seven mutational signatures were evaluated for each sample by minimizing the constrained linear function:


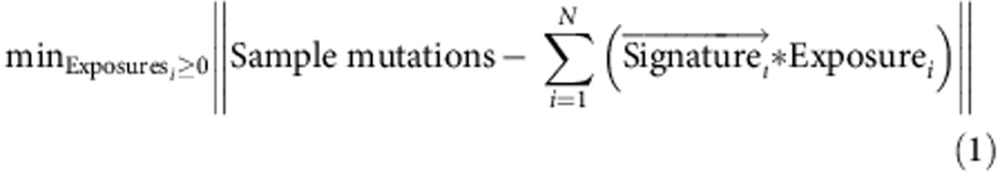


Here 
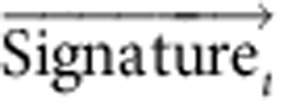
 represents a vector with 96 components (corresponding to the six somatic substitutions and their immediate sequencing context) and Exposure_*i*_ is a non-negative scalar reflecting the number of mutations contributed by this signature. *N* reflects the number of signatures found in the sample and all possible combinations of consensus mutational signatures for *N* between 1 and 7 were examined for each sample. This resulted in 1,285,623 solutions per sample and a model selection was applied to select the optimal solution. The model selection framework excludes any solution in which a mutational signature contributes <3% of the somatic mutations or <50 somatic mutations. Exceptions were made for signatures 1A and 5 as these are believe to reflect on-going endogenous mutational processes that continuously contribute very low numbers of somatic mutations^19^. Further, the model selection framework selects the solution that optimizes the correlation between the original pattern of somatic mutations and the one based on refitting the sample with consensus mutational signatures such that each additional signature should significantly improve the Pearson correlation. The final solution for each sample contained not more than five mutational signatures.

### Identification of subclones and evolutionary trees

To unravel the spatial and temporal dynamics of tumour subclonal diversity within a patient, we modelled the observed SNV allele frequencies with a Bayesian Dirichlet process^12^,^51^.

Clusters of mutations were identified in *n* dimensions, where each dimension represented the allele frequencies for a given sample (adjusted for copy number and tumour ploidy). These mutation clusters facilitated the detection of clonal and subclonal cell populations in the primary and metastatic samples within a patient. This analysis permitted derivation of the temporal and spatial ordering of clonal subtype heterogeneity for each of the tumours for each of the four patients. The *n*-dimensional posterior mutation density (where *n* is the number of samples from each patient) was estimated from the Bayesian Dirichlet process using the R package ks. Local peaks in density were identified and the ‘basin of attraction’ of each peak was estimated by finding the point of minimum density between each pair of peaks. Each mutation was then assigned to the peak in whose basin of attraction it was most likely to fall, using the posterior distribution obtained from the Dirichlet process. For each patient, this process was applied to WGS data, and then repeated using the deep targeted sequencing data (Supplementary Figs 2 and 7) to refine subclonal frequencies.

### Identification of mutation clusters in whole blood

For each of the clusters identified by the Bayesian Dirichlet process, a number of mutations were subjected to targeted ultra-deep sequencing in both tissue and whole-blood samples. For the whole-blood samples, the number of wild type, mutant and ‘other’ reads at each mutation loci were found. ‘Other’ reads refers to those reads reporting neither the wild-type nor mutant allele. These were used as a background model to estimate the rate of misreads at each locus. If a mutation was not present in a blood sample, the expected number of mutant reads is half the number of ‘other’ reads. A Poisson test with a rate ratio of 0.5 was used to test this hypothesis on the sum of mutant and ‘other’ reads from each mutation cluster.

### Validation of tumour evolution trees using SVs

For patients 299, 498 and 001, we sought evidence that the tree topology derived from the SNVs (Fig. 1a–c) were supported by SVs. For many nodes in the tree, this simply required calculating the presence or absence of a SV in a sample. However, for patients 299 and 498, the subclonality observed in some of the samples meant that it was necessary to look at the SV allele frequencies to confirm the tree topology. This also had the added benefit of being able to assign SVs to all branches in the tree.

For each of the somatic structural variation breakpoints *b*ε1...*B*, we calculated the SV allele frequency:


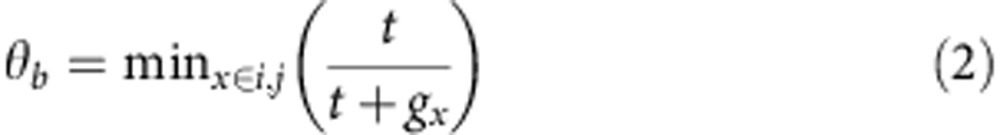


where *i* and *j* represented the genomic locations of the ‘left’ and ‘right’ hand sides of the break, respectively, *t* was the number of paired-end reads supporting (but with neither read overlapping) the break and *g* was the number of normal genomic reads spanning the break at location *x*.

During analysis of these SV allele frequencies, no adjustment was made for tumour cellularity or ploidy thus all values are reported as raw frequency estimates. SVs were assumed to occur on a single chromosome and so all SVs with frequencies above this were removed from analysis. In addition, all breaks residing in regions showing loss of heterozygosity from copy-number calling were also removed.

Of the 119 SVs identified across the two samples in patient 299, 93 were shared between the primary (bT) and metastasis (aM) confirming that these tumours were derived from a common ancestor (Supplementary Fig. 6, confirming branch A), 3 were unique bT and 23 were unique to aM also confirming the presence of two subclonal populations of cells in bT, one of which gave rise to aM (Supplementary Fig. 6, confirming branches B and D). According to the tree topology, the SVs unique to bT should appear at frequencies subclonal in roughly 64% of tumour cells. The SV allele frequencies unique to the primary show a mean frequency of 0.23 (0.19–0.28, 95% confidence interval (CI); Supplementary Fig. 6). Using the estimated tumour cellularity of 0.74 and the fact that bT was diploid, the proportion of cells containing these variants could be estimated from SV allele frequencies as 0.23/(0.74/2)=0.62 or 62% (51–76, 95% CI). This is approximately equivalent to the 64% estimated from the SNV allele frequencies thus confirming the overall tree topology and subclonal frequency estimates.

For patient 498, we constructed a matrix containing the presence/absence of SVs across all five samples bT (fresh primary), aR (surgical bed metastasis), aM (sacral metastasis), cM (iliac metastasis) and fTM2 (castrate-resistant iliac metastasis). Given the tree topology identified in Fig. 1b, we assigned SVs to all branches in the tree, except those connected to the node containing fTM2, which we collapsed into a single branch due to subclonality. One hundred and ninety-four (92%) of the SVs mapped onto the tree topology, with only 18 showing disagreement, suggesting that the tree topology identified using the SNVs was adequate. To tease apart the SVs belonging to the subclones in fTM2 and confirm the subclonal fraction estimates, we performed Partitioning Around Medoids clustering^48^ (*k*=2) on the observed SV allele frequencies unique to fTM2 (Supplementary Fig. 6). The clustering identified medoids at 0.11 and 0.06. Given the estimated tumour cellularity of 0.52 and the fact the fTM2 was triploid, the subclonal populations were estimated to make up 0.11/(0.52/3)=0.62 or 62% (0.49–0.64, 95% CI) and 0.06/(0.52/3)=0.33 or 33% (0.1–0.35, 95% CI). These estimates are approximately equivalent to the estimates in Fig. 1b thus confirming the overall tree topology and subclonal frequency estimates.

### Reconstruction of the tumour evolution tree (patient 177)

For patient 177, a cluster of SNVs unique to the metastasis were found at an (impossible) frequency greater than 100% after adjustment for copy number, tumour cellularity and ploidy suggestive of an error somewhere in estimation (Supplementary Fig. 2c). The original tumour cellularity was estimated at 23%; however, the SV allele frequencies showed a cluster of frequencies at >23% confirming the original estimate was incorrect (Supplementary Fig. 3). Using the maximum peak of the observed density distribution for the SV allele frequencies, we estimated the tumour cellularity to be 79%. Using this new estimate and clusters of observed allele frequencies from both the SNVs and SVs, we manually reconstructed a tree topology which fit the data. A large number of tree topologies were considered before one which best fit the data was found. The following provides a rationale for the reconstruction. A likely explanation for the discrepancy between the maximum observed SV allele frequencies (0.40) and the maximum observed SNV allele frequencies (0.15) is as follows: there was an acquisition of a number of SVs early in the evolution of the tumour (Branch A); followed by an early wave of metastatic spread (Branch C); the primary continued to evolve acquiring a number of SNVs (Branch B); this was followed by a second wave of metastasis with this population of cells making up the minority of the subclonal fraction, hence the low overall SNV allele frequencies (Branch E); the primary tumour continued to evolve before being sampled (Branch D). The SVs shared between the metastasis and primary tumour revealed two distinct mutations clusters, one representing the clonal SVs at 0.40, and another at 0.08. Given the tree topology, this second cluster must have occurred on branch B, thus providing an estimate for the subclonal fraction of *x=*0.08/(0.79/2)=0.20 or 20% (Supplementary Fig. 3). The SVs unique to the metastasis showed a single cluster (Supplementary Fig. 3) that was likely an indistinguishable combination of subclonal fraction *x* and the remaining tumour cells. The SNVs unique to the metastasis did not reveal a distinct cluster at 0.08, suggesting few SNVs occurred on branch E. Therefore these SNVs must have occurred in the remaining tumour fraction. Two clusters were observed in these remaining SNVs at 0.14 and 0.17 (identified using PAM clustering). This suggested a division of the remaining fraction into a further two subclonal populations (branches F and G). The fractional estimate of these two subclones were y=0.14/(0.79/2)=0.35 and z=0.17/(0.79/2)=0.43. This resulted in the final tree topology observed in Fig. 1c. SNVs were assigned to branches in the tree using PAM clustering assignments and SVs were placed on branches by assigning each SV to its nearest subclonal fraction mean frequency estimate. Theoretically it would be possible to confirm the subclonal breakdown of the metastasis if there were mutually exclusive mutations on single sequencing reads which were unique to each subclonal population. However, no such mutations were in close genomic proximity rendering targeted capture sequencing unable to validate the subclonal reconstruction.

### Identification of mutations in known cancer drivers

We compiled a list of known cancer drivers by combining those genes in the COSMIC gene census^52^ and those recently annotated by Tamborero *et al*.^53^ We annotated all mutations identified via WGS using ANNOVAR^54^ and filtered these considering only those appearing in the cancer gene list. Any mutation that was predicted to be deleterious by at least two of SIFT, Polyphen2, MutationTaster, MutationAssessor was considered a putative driver mutation. For copy-number variations, we used the Mann–Whitney *U*-test to test for significantly different gene copy-numbers between primary and metastatic samples across our cohort. Only AR and MSN showed significant (false discovery rate-corrected *P* value<0.05) copy-number changes using this approach.

## Author contributions

M.K.H.H., S.L., K.P., D.T. and N.K. performed the experiments. G.M., D.C.W., P.V.L., M.C., S.M., F.M., L.B.A., S.L., A.L., C.S., H.N. and I.H. performed the data analysis. M.K.H.H., C.M.H., N.S., M.K., A.J.C., N.M.C., P.M.P., J.S.P., A.R. and X.C. obtained metastatic cohort samples. A.Y.W., D.N. and V.G. obtained localized cohort samples. C.M.H., N.M.C. and N.R. supervised the research. G.M. and C.M.H. designed the experiments, performed the analysis and wrote the manuscript.

## Additional information

**Accession codes:** All sequence data from tumour samples have been deposited into the EGA Sequence Read Archive under accession number EGAS00001000942.

**How to cite this article:** Hong, M. K. H. *et al*. Tracking the origins and drivers of subclonal metastatic expansion in prostate cancer. *Nat. Commun.* 6:6605 doi: 10.1038/ncomms7605 (2015).

## Supplementary Material

Supplementary Information Supplementary Figures 1-9 and Supplementary Tables 1-2
