# Supplementary material for: Tracking the origins and drivers of subclonal metastatic expansion in prostate cancer
Source: Nat Commun. 2015 Apr 1;6:6605. doi: 10.1038/ncomms7605 (PMC4396364; doi:10.1038/ncomms7605)
Supplement: Supplementary Information — Supplementary Figures 1-9 and Supplementary Tables 1-2 [file ncomms7605-s1.pdf]

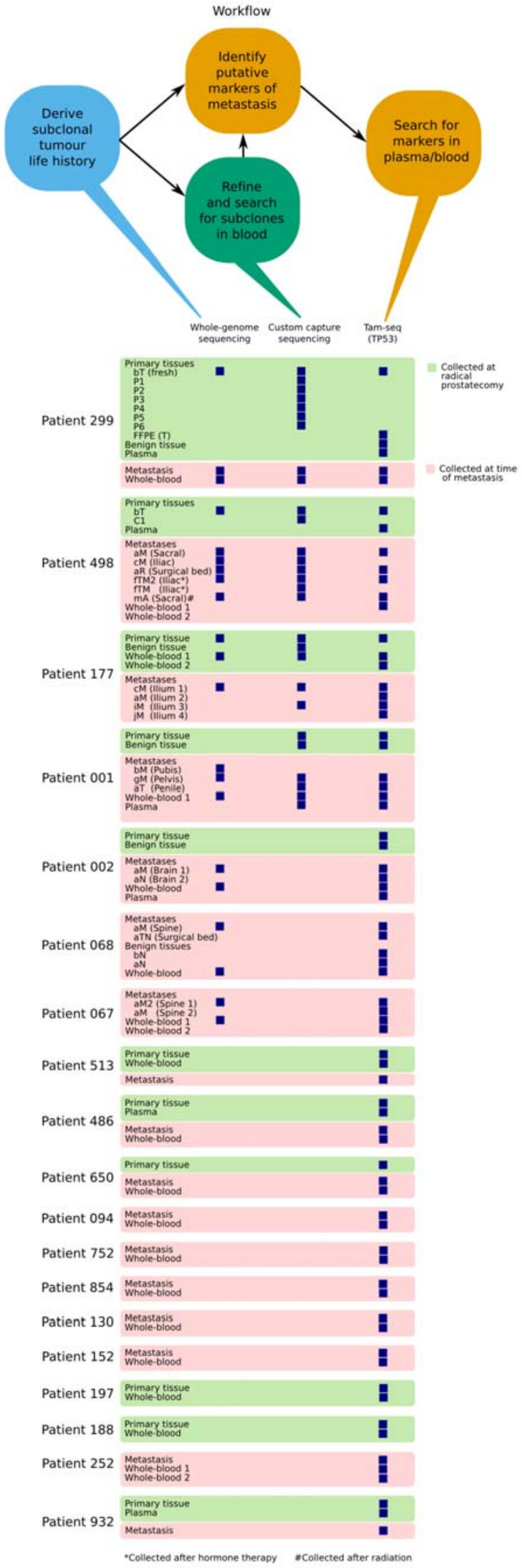

Supplementary Figure 1: A schematic indicating the sequencing strategy for the study and indicators of metastatic cohort samples interrogated.

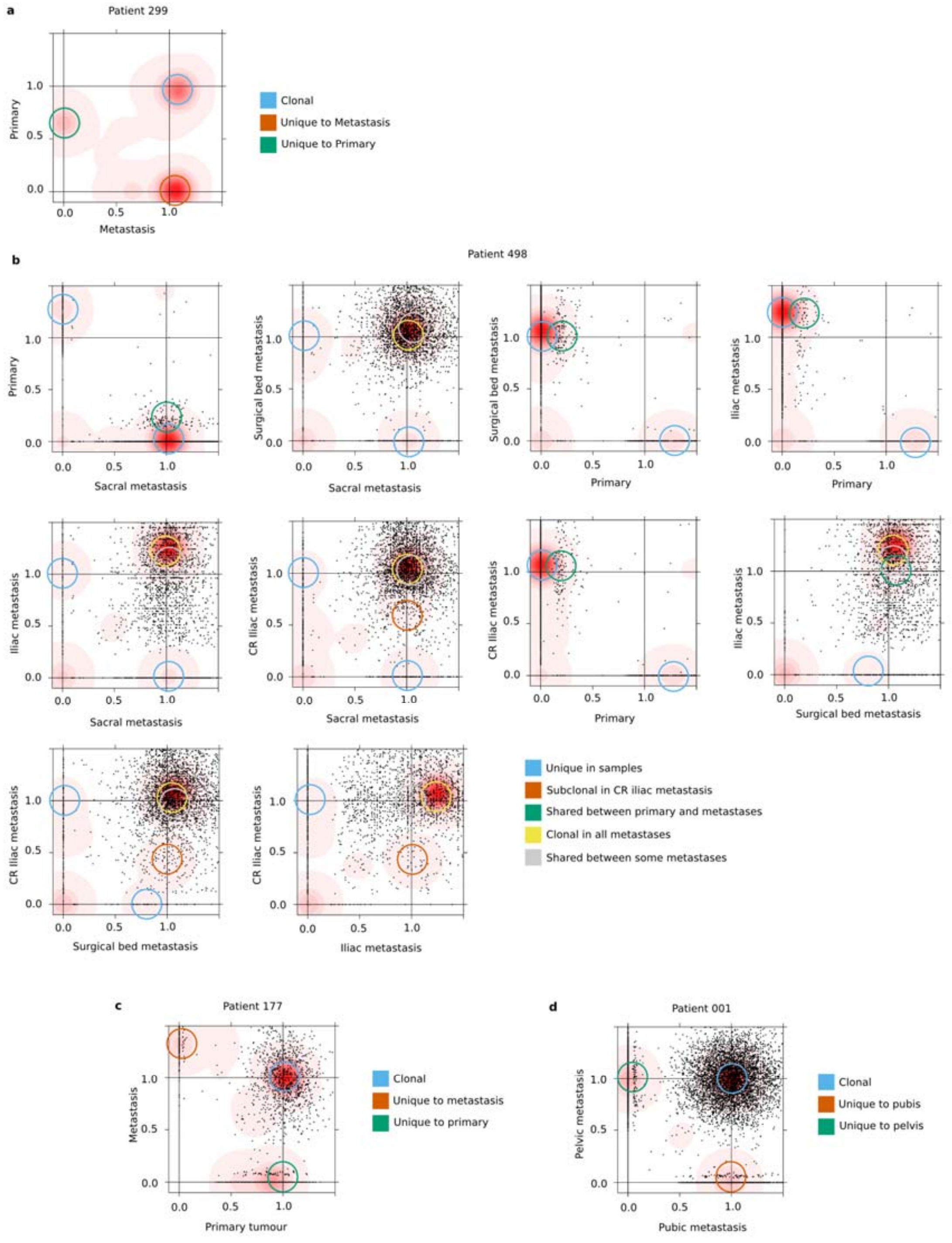

Supplementary Figure 2: Variant allele frequency plots showing the shared, private and subclonal mutations identified using whole-genome sequencing information for patient 299, 498, 177 and 001.

The 2D contour plots represent the allele frequencies of clusters of mutations identified by the Dirichlet process for pairs of samples interrogated by whole-genome sequencing. Each plot shows the mutation allele frequencies adjusted for copy-number and tumour cellularity between two samples (black dots). Deeper red colour represents higher posterior probability of a cluster. The circles around the clusters indicate the pools of mutations used for selection of variants for deep targeted resequencing.

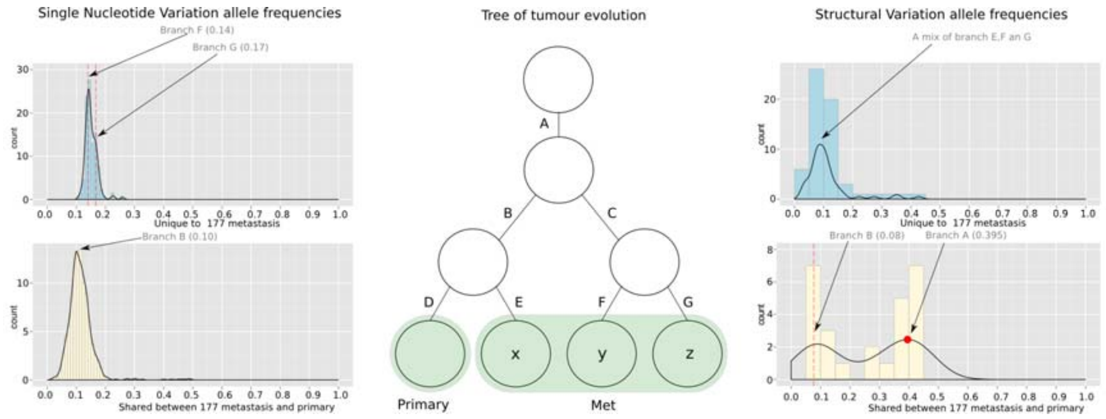

Supplementary Figure 3: Single-nucleotide and structural variation allele frequency distributions for patient 177.

The SNV (left) and SV (right) allele frequency histograms and density plots (black line) for variations unique to the metastasis in patient 177 (top) and variations shared between the metastasis and primary tumour (bottom). The dotted red lines represent the medoids identified by PAM clustering. The tree represents the evolution of the tumour and the allele density peaks representing various tree branches are marked. The red dot represents the density point used for estimating tumour cellularity.

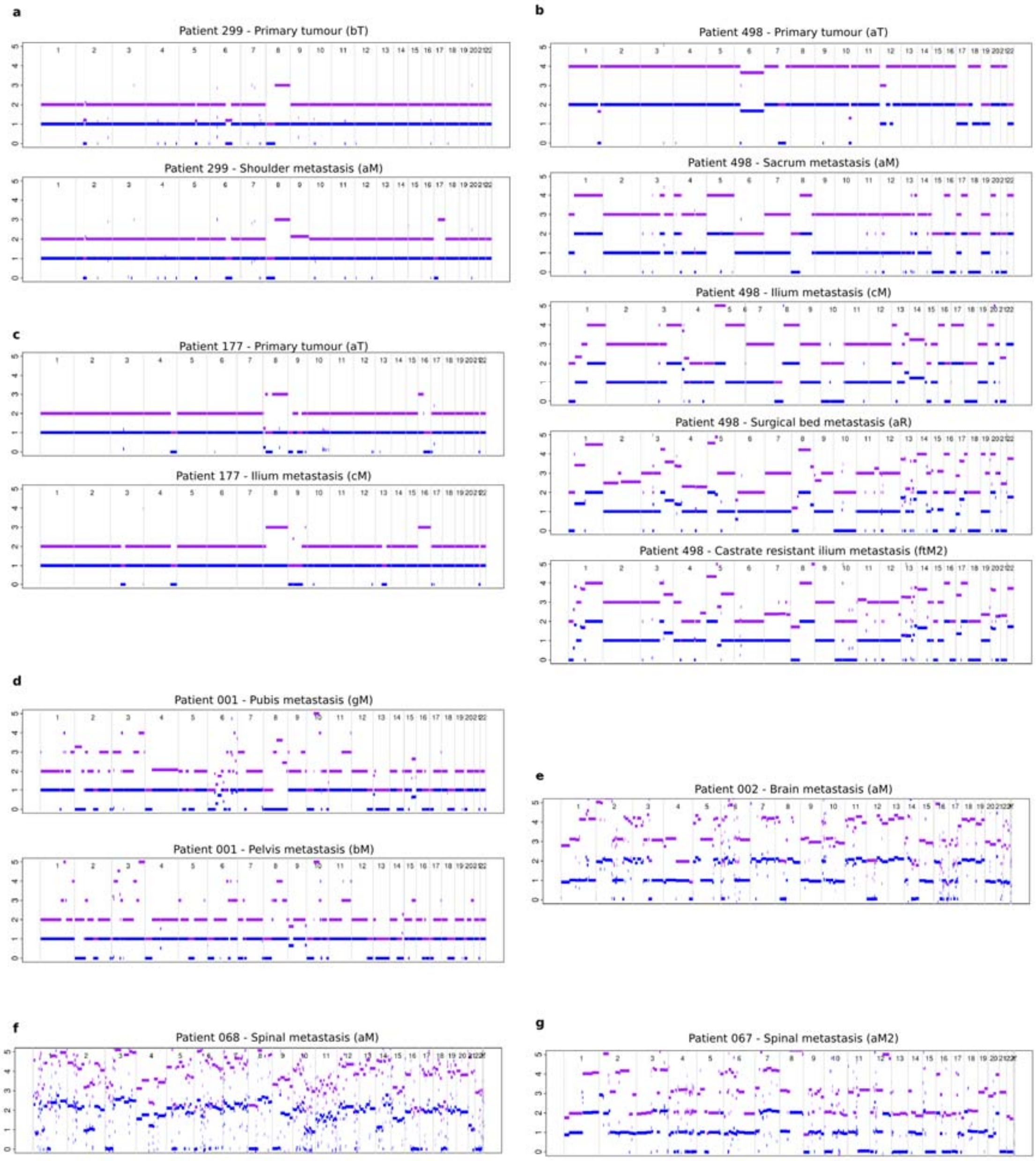

Supplementary Figure 4: Genome-wide copy-number profiles for samples from patients 299, 498, 177, 001, 002, 067 and 068.

Purple lines represent the total copy number, and blue the minor allele copy number.

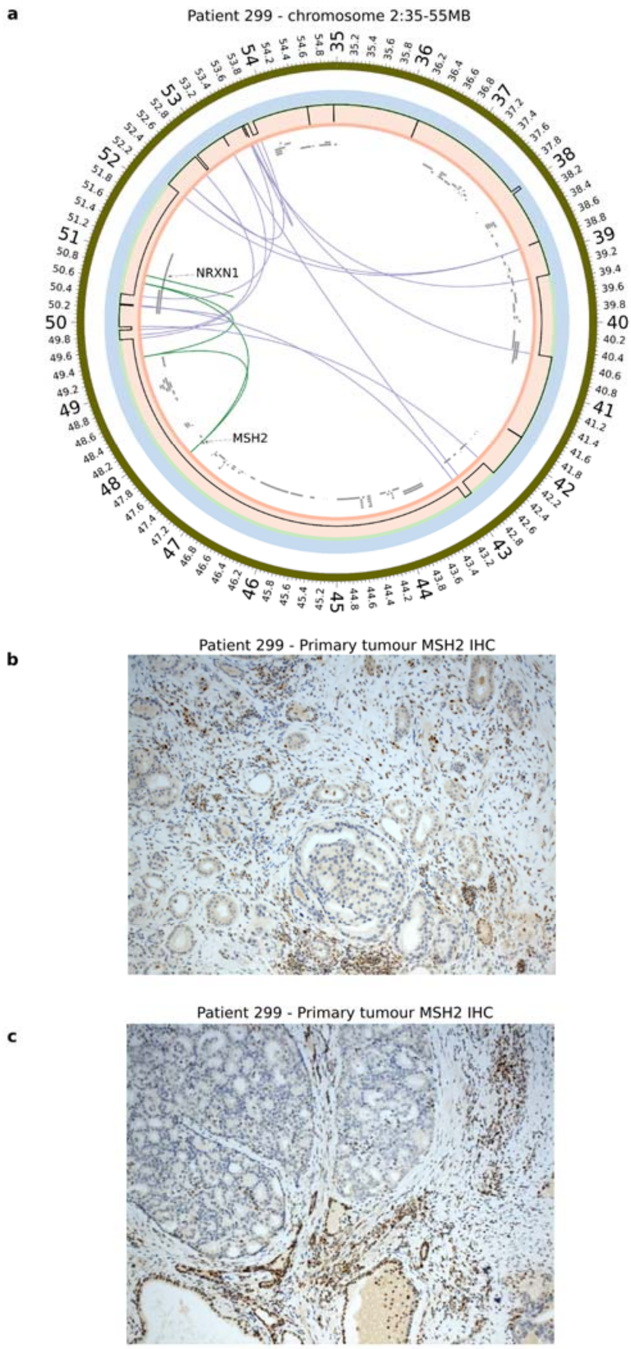

Supplementary Figure 5: Structural rearrangement and loss of MSH2 locus in patient 299. **a)** A circos plot depicting structural rearrangements of the MSH2 locus in patient 299 containing genomic coordinates, copy-number (black line, shading blue=gain, green=normal, orange LOH, red=loss), genes (grey bars), and structural rearrangements colour coded by chromoplexy event. **b)** MSH2 IHC staining showing no staining of tumour cells and positive staining of surrounding lymphocytes and **c)** positive staining of adjacent benign glands.

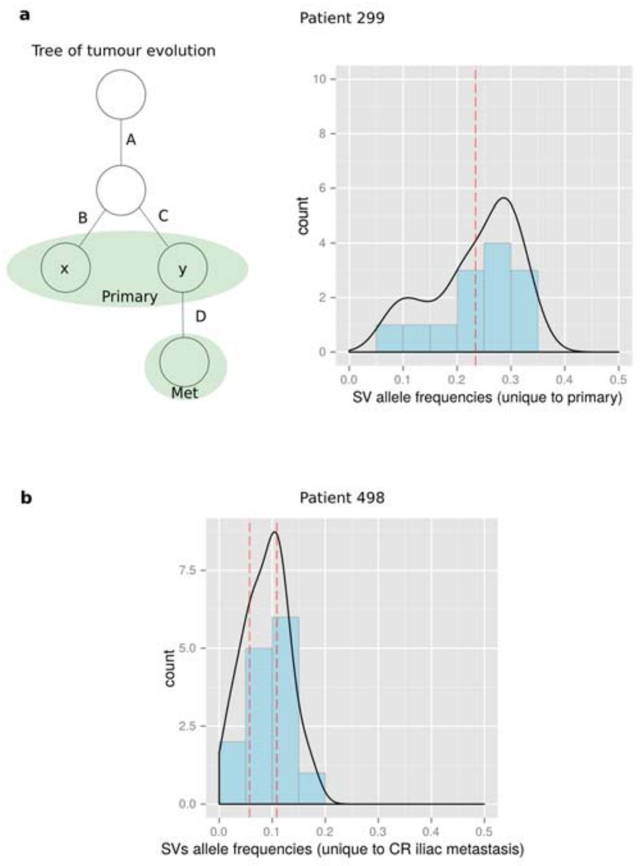

Supplementary Figure 6: Structural variation allele frequencies for patient 299 and 498. The histograms and density plots (black line) show the allele frequencies observed for SVs for the primary tumour from patient 299 (a) and the castrate resistant iliac metastasis from 498 (b). The dotted red lines represent the medoids of identified by PAM clustering and are representative of the subclonality observed in these samples. The tree for patient 299 is indicated to demonstrate that the primary tumour consists of a mixture of two clones.

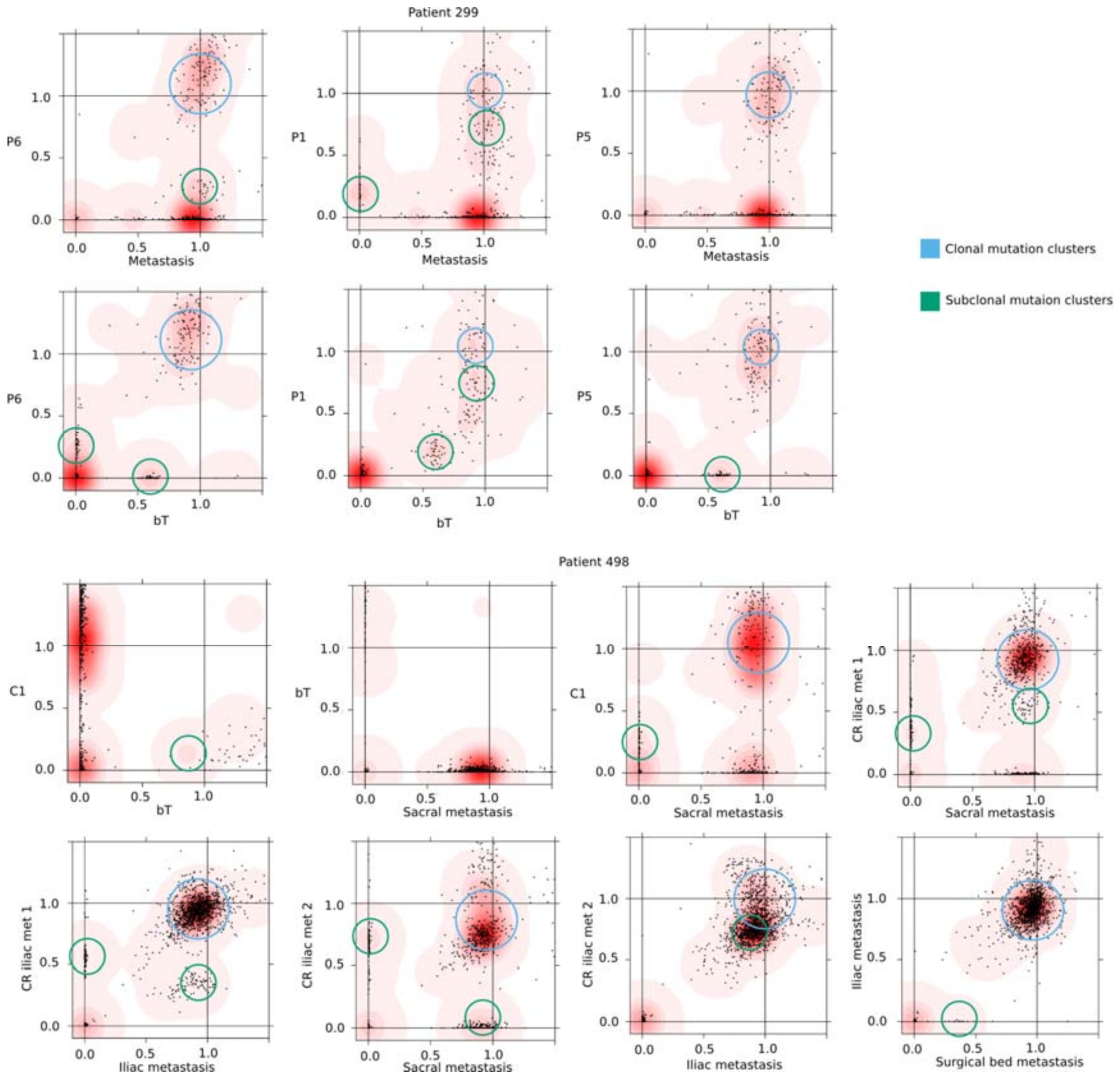

Supplementary Figure 7: Variant allele frequency plots showing the shared, private and subclonal mutations identified using deep, targeted resequencing information for patients 299 and 498.

The 2D contour plots represent the allele frequencies of clusters of mutations identified by the Dirichlet process for pairs of samples interrogated by whole-genome sequencing. Each plot shows the mutation allele frequencies adjusted for copy-number and tumour cellularity between two samples (black dots). Deeper red colour represents higher posterior probability of the cluster. The circles on the plots indicate mutations which appear clonal (shared between samples) or subclonal (private to a proportion of cells in the sample). These clusters form the basis of the sublconal estimates indicated in Figure 1.

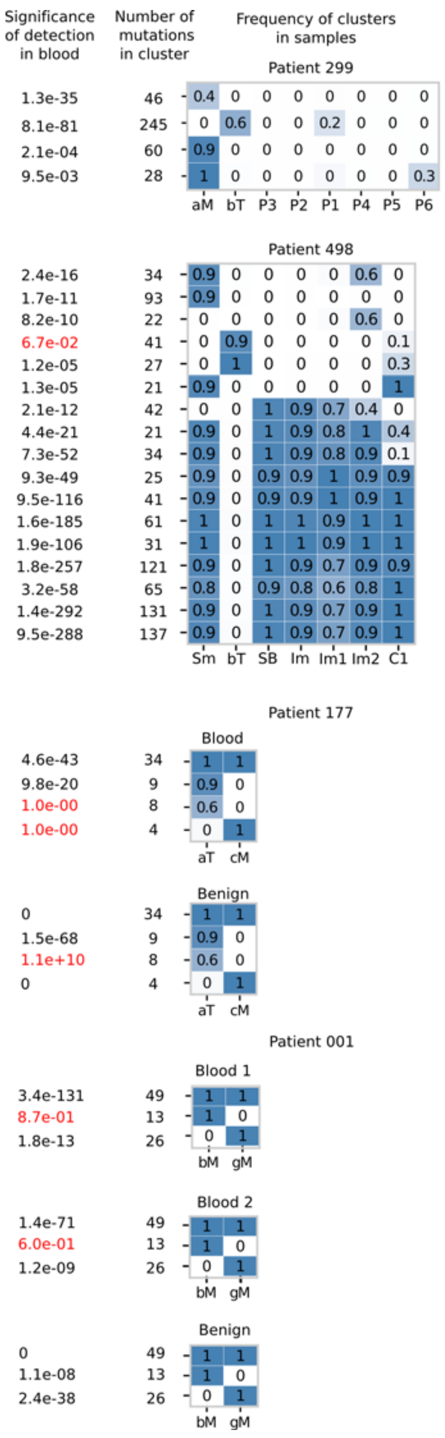

Supplementary Figure 8: Mutation clusters representative of tumour clones detected in deep resequencing of whole-blood from patients 299, 498, 177 and 001.

The matrices show the allele frequency of different mutation clusters for various samples. For each of the mutation clusters, the number of mutations and the FDR corrected significance of the Poisson test used for detecting the mutation clusters above background sequencing noise in the whole-blood and benign tissue samples is listed. 498: Sm = sacral, SB=surgical bed, Im=iliac, Im1/2=CR iliac. 177: cM=metastasis. 001: bM=pubic metastasis, gM=pelvic metastasis.

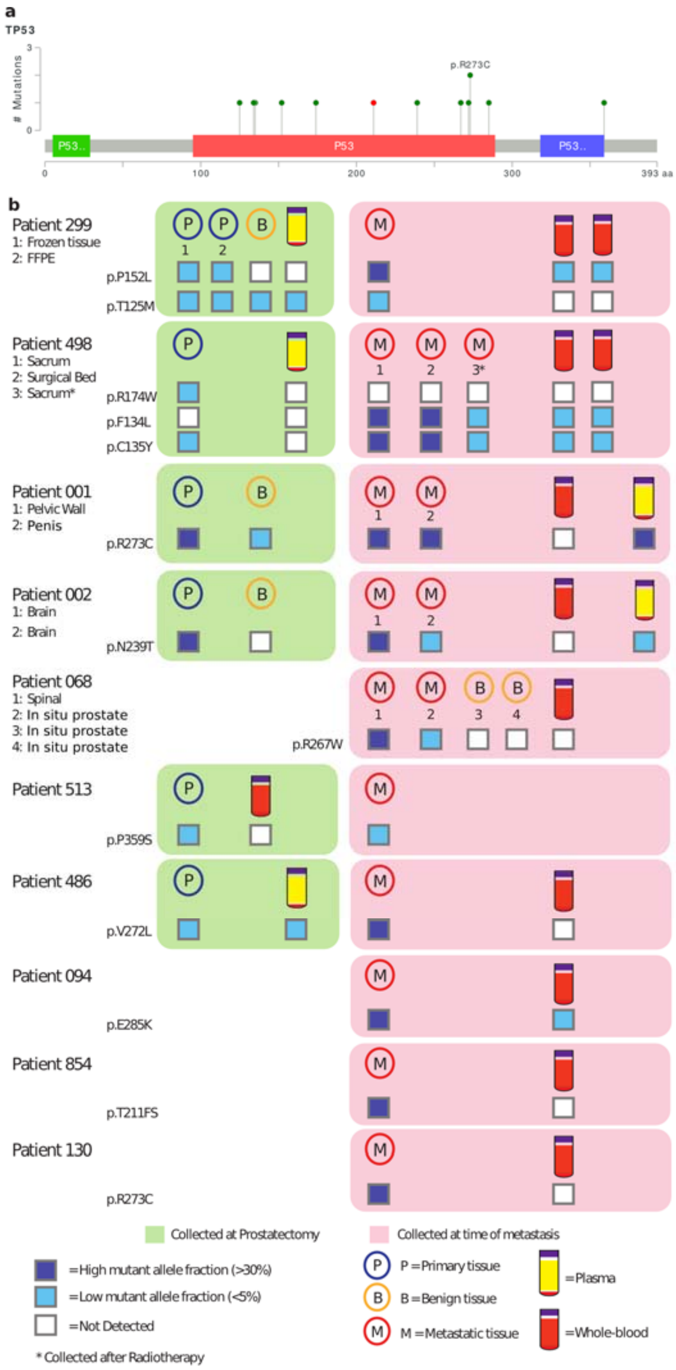

Supplementary Figure 9: TP53 mutations identified via tam-seq.

a) A schematic indicating the location of each of the identified TP53 mutations within the protein. Mis-sense mutations are shown in green and frame-shift in red. **b)** The detection results for the application of targeted amplicon sequencing of TP53 across 72 samples. Only patients with a TP53 mutation are listed (n=10), the remaining nine patients who were negative for TP53 mutations are not shown.

PatientID	001		002	067	068	177		299		498				
Age at diagnosis	73		77	60	71	53		64		71				
Gleason grade (primary tumour)	4+5		4+5	3+4	4+5	4+3		4+5		3+4				
Tumour Sample ID	001bM	001gM	002aM	067aM2	068aM	177aT	177cM	299bT	299aM	498bT	498aR	498aM	498cM	498fTM2
Tumour coverage	51.5	58.9	50.3	56.1	57.3	58.7	97.7	54.4	39.7	41.7	25.2	29.4	20.7	31.9
Extimated tumour purity (%)	49	46	86	67	58	68	23	71	74	36	81	82	23	52
Average ploidy	2.26	2.22	3.71	3.13	3.8	2.05	1.89	2.09	2.15	3.83	2.77	3.05	3.25	2.87
SNVs	7237	7251	6581	6369	3709	1680	1321	49192	28983	692	3167	3354	2902	3292
Structural rearrangements	286	291	175	188	220	81	153	96	100	49	124	112	117	144

Supplementary Table 1: Summary of clinical characteristics and sequencing results of patients subjected to whole-genome sequencing.

Sample	Primary(P)/ Metastasis(M)/ Benign(B)	Chr	Position	Reference	Mutation	AF (weighted average)
002_blood	M	17	7577565	T	G	0.04%
002_plasma	M	17	7577565	T	G	1.54%
002_tissue01	M	17	7577565	T	G	76.09%
002_tissue02	M	17	7577565	T	G	0.97%
002_tissue03	P	17	7577565	T	G	45.29%
002_tissue04	P	17	7577565	T	G	0.00%
014_blood01	M	17	7579422	G	A	0.26%
014_blood01	M	17	7579421	G	A	0.46%
014_blood01	M	17	7577121	G	A	0.25%
014_plasma	M	17	7579422	G	A	0.36%
014_plasma	M	17	7579421	G	A	0.64%
014_plasma	M	17	7577121	G	A	30.00%
014_tissue01	M	17	7579422	G	A	0.20%
014_tissue01	M	17	7579421	G	A	0.16%
014_tissue01	M	17	7577121	G	A	37.65%
014_tissue02	P	17	7579422	G	A	0.78%
014_tissue02	P	17	7577121	G	A	48.09%
014_tissue02	P	17	7579421	G	A	1.02%
014_tissue03	B	17	7579422	G	A	1.36%
014_tissue03	B	17	7577121	G	A	1.66%
014_tissue03	B	17	7579421	G	A	1.20%
014_tissue04	M	17	7577121	G	A	35.45%
014_tissue04	M	17	7579421	G	A	0.18%
014_tissue04	M	17	7579422	G	A	0.20%
068_blood	M	17	7577139	G	A	0.18%
068_bloodrep_2	M	17	7577139	G	A	0.19%
068_Tissue02	M	17	7577139	G	A	32.98%
068_Tissue03	B	17	7577139	G	A	0.17%
068_Tissue04	B	17	7577139	G	A	0.19%
068_Tissue05	M	17	7577139	G	A	0.65%
094_blood	M	17	7577085	C	T	0.00%
094_bloodrep_2	M	17	7577085	C	T	0.15%
094_tissue	M	17	7577085	C	T	33.02%
130_blood	M	17	7577121	G	A	0.24%
130_bloodrep_2	M	17	7577121	G	A	0.24%
130_tissue	M	17	7577121	G	A	60.96%
299_blood01	M	17	7579313	G	A	0.19%
299_blood01	M	17	7578475	G	A	0.30%
299_blood01rep_2	M	17	7578475	G	A	0.36%
299_blood01rep_2	M	17	7579313	G	A	0.20%
299_plasma	P	17	7579313	G	A	0.16%
299_plasma	P	17	7578475	G	A	0.12%
299_tissue01	B	17	7579313	G	A	0.20%

299_tissue01	B	17	7578475	G	A	0.22%
299_tissue04	P	17	7579313	G	A	0.43%
299_tissue04	P	17	7578475	G	A	0.38%
299blood_02	M	17	7578475	G	A	0.25%
299blood_02	M	17	7579313	G	A	0.28%
299tissue_02	P	17	7579313	G	A	0.23%
299tissue_02	P	17	7578475	G	A	0.32%
299tissue_03	M	17	7578475	G	A	66.49%
299tissue_03	M	17	7579313	G	A	3.53%
486_blood	M	17	7577124	C	G	0.00%
486_bloodrep_2	M	17	7577124	C	G	0.01%
486_plasma	P	17	7577124	C	G	0.26%
486_tissue01	P	17	7577124	C	G	0.35%
486_tissue02	M	17	7577124	C	G	58.72%
498_blood01	M	17	7578528	A	T	0.31%
498_blood01	M	17	7578526	C	T	0.34%
498_blood01	M	17	7578410	T	A	0.07%
498_blood01rep_2	M	17	7578526	C	T	0.29%
498_blood01rep_2	M	17	7578410	T	A	0.10%
498_blood01rep_2	M	17	7578528	A	T	0.41%
498_blood02	M	17	7578526	C	T	0.57%
498_blood02	M	17	7578528	A	T	0.54%
498_blood02	M	17	7578410	T	A	0.29%
498_plasma	P	17	7578528	A	T	0.06%
498_plasma	P	17	7578526	C	T	0.19%
498_plasma	P	17	7578410	T	A	0.13%
498_tissue01	P	17	7578528	A	T	0.05%
498_tissue01	P	17	7578410	T	A	0.89%
498_tissue01	P	17	7578526	C	T	0.31%
498_tissue02	M	17	7578526	C	T	24.81%
498_tissue02	M	17	7578410	T	A	0.02%
498_tissue02	M	17	7578528	A	T	60.45%
498_tissue03	M	17	7578410	T	A	0.06%
498_tissue03	M	17	7578528	A	T	65.35%
498_tissue03	M	17	7578526	C	T	27.91%
498_tissue04	M	17	7578410	T	A	0.08%
498_tissue04	M	17	7578526	C	T	0.58%
498_tissue04	M	17	7578528	A	T	0.70%
513_blood	P	17	7573952	G	A	0.18%
513_bloodrep_2	P	17	7573952	G	A	0.17%
513_tissue01	P	17	7573952	G	A	1.70%
513_tissue02	M	17	7573952	G	A	0.14%
CamP_FFPE_11	P	17	7579422	G	A	6.65%

Supplementary Table 2: Summary of TP53 mutant allele determined using tam-seq.
